# The Neurophysiological Effects of Craniosacral Treatment on Heart Rate Variability: A Systematic Review of Literature and Meta-Analysis

**DOI:** 10.7759/cureus.64807

**Published:** 2024-07-18

**Authors:** Andrew C Cook, Anna E Egli, Nathan E Cohen, Kyrie Bernardi, Min Y Chae, Brandon A Kapalko, Sunni A Coyne, Randy Scott

**Affiliations:** 1 College of Osteopathic Medicine, Lake Erie College of Osteopathic Medicine - Bradenton, Jacksonville, USA; 2 College of Osteopathic Medicine, Lake Erie College of Osteopathic Medicine - Bradenton, St. Augustine, USA; 3 College of Osteopathic Medicine, Lake Erie College of Osteopathic Medicine - Bradenton, Daytona Beach, USA; 4 Regional Dean, Lake Erie College of Osteopathic Medicine - Bradenton, Jacksonville, USA

**Keywords:** autonomic nervous system dysfunction, craniosacral therapy, cv4, heartrate variability, high-frequency power, low-frequency power, neurophysiology, osteopathic cranial manipulative medicine, parasympathetic stimulation, sympathetic overactivity

## Abstract

Craniosacral treatment (CST) is an osteopathic technique grounded in the assumption that there is an intrinsic, fine movement of the cerebrospinal fluid. This rhythmic movement can be utilized for diagnostic and therapeutic purposes by palpation and manipulation of the skull, spine, and associated connective tissues. Therapeutic benefit is likely due to action on the autonomic nervous system (ANS), specifically through the vagus nerve. Current literature on the neurophysiological effects of CST is limited, which has contributed to controversy regarding its effectiveness. Heart rate variability (HRV) as a measure of cardiovascular stress and autonomic system activity is thus proposed as a tool to evaluate the neurophysiologic effects of CST. HRV can be analyzed in two different bands, high-frequency (HF) and low-frequency (LF) power associated with a parasympathetic and sympathetic response. In this meta-analysis, we provide a brief introduction to CST, analyze three primary studies, and summarize the therapeutic benefits and pitfalls of this alternative treatment on the ANS. A significant negative HF standardized mean difference after CST was observed; standardized mean difference = -0.46; 95% CI (-0.79,-0.14). No significant effect on LF power was observed. We conclude that CST does provide a moderate short-term increase in parasympathetic activity. These findings suggest that CST may be used to treat patients with an overactive sympathetic state. Further studies should be conducted for comparison against a control group to eliminate the possibility of a placebo effect and to elucidate long-term effects.

## Introduction and background

The technique of craniosacral treatment (CST) originated from John E. Upledger, DO who discovered the rhythmic impulse of cerebrospinal fluid (CSF) upon grasping the dura mater during surgery for a patient who had been infected with Echinococcus [1]. While grasping the dura mater, he saw and felt the expansion of the dura mater in a consistent cycle. With no established explanation for the phenomenon he witnessed, Dr. Upledger dedicated research to the development of his theory, the Pressurestat model. Dr. Upledger further discovered that nerve tracts are found to originate from cranial sutures and travel to the choroid plexus of the ventricular system. His theory suggested that the choroid plexus, responsible for producing CSF and causing volume expansion within the ventricular system, transmits this expansion to the cranial sutures. Nerves originating from the cranial sutures where the dura mater is fused with the periosteum conduct this expansion. When a threshold of “stretch” is achieved, these nerves send negative feedback to inhibit CSF production. On the contrary, lack of CSF will decrease the distance between sutures promoting CSF production. It was later discovered that the cranial rhythmic impulse (CRI) is not an inherent function of the brain but rather a cerebrovascular wave pulse [2]. When the heart undergoes systole, blood propulsion distends cerebral arteries, compressing the ventricles and sending CSF into the subarachnoid space and spinal canal. With this mechanism in mind, it has been observed that the normal rate of CRI is 8-10 cycles per minute [1]. When disease is present in the body, this rate may vary. For example, Dr. Upledger mentions the research of Dr. Bunt and Dr. Allen, MD, who discovered that patients with idiopathic hydrocephalus have an irregular CRI rate of four cycles per minute [1]. Additionally, patients who were paralyzed secondary to spinal cord injury were found to have elevated CRI rates in areas below the injury but normal rates above [1]. Changes in the rate of CRI indicate areas of increased sympathetic activity related to pathology including inflammation, disease, and injury. CST seeks to restore balance to the autonomic nervous system (ANS) [3]. For example, one technique in practice is compression of the fourth ventricle (CV4) [4]. The CV4 technique involves the patient lying supine with the practitioner placing his or her thenar eminence along the lateral protuberances of the squamous occiput. The practitioner facilitates the extension of the primary respiratory movement by applying a medial compression to the occiput in synchrony with a cephalad traction. The practitioner resists flexion of the primary respiratory movement and holds this position until a still point of undetectable CRI is felt. This manipulation is concluded when the CRI is again palpable. Detection of the still point is an indication of ANS balance. Measurement of heart rate variability (HRV) is a proposed mechanism to quantify the effect of this osteopathic technique on the ANS [4].

HRV is defined as the variation in time intervals between consecutive heartbeats measured through R-R intervals [5]. In the clinical setting, it is an assessment of the adaptability of the heart to stressors of emotional, physiological, pathological, environmental, and lifestyle significance by the ANS. The ANS is divided into the sympathetic and parasympathetic systems, both of which play vital roles in homeostasis and adaptation to internal and external stimuli [5]. An imbalance of these systems can often be a sign of pathology, with one system competing with the effects of the other. Instances of this phenomenon can manifest as heart block, syncope, or orthostatic hypotension, which occur when the parasympathetic system is overly active and promotes bradycardia [6]. Conversely, hypertension, vascular disease, and myocardial infarctions are the product of an overly activated sympathetic state with increased heart rate but decreased HRV. A decrease in HRV is associated with morbidity as it yields risk of arrhythmia and an inflammatory state that is less adaptable to stressors [5]. The parasympathetic system is largely controlled by the vagus nerve (CNX), which innervates the sinoatrial and atrioventricular nodes of the heart, myocardium, lungs and gastrointestinal system. The premise of CST is soft pressure manipulations to stimulate parasympathetic tone and reestablish balance throughout the ANS [3].

We sought to compose a literature analysis to determine the statistical association between CST and the neurophysiology of the ANS. While there are some individual studies investigating this, none have summarized the effect size in a systematic review. This will be useful as a foundation to determine if CST is effective in altering a response in the ANS. Clinically, this study can prompt future research into specific treatment protocols targeting autonomic somatic dysfunctions. Criticisms of CST include debate on the reliability of palpating the craniosacral rhythm as well as the lack of quality randomized-controlled studies on this technique [7,8]. HRV data was measured with two metrics, high-frequency (HF) and low-frequency (LF) power [9]. HF power is the frequency range associated with respiratory sinus arrhythmia and is measured in the range of 0.15-0.40 Hz. An increase in HF power is associated with an increased parasympathetic state. Meanwhile, LF power is measured in the range of 0.04-0.15 Hz and is associated with both parasympathetic and sympathetic activity, but primarily the latter. The meta-analysis we conducted examined HF and LF power data before CST and immediately after. Results were summarized using standardized mean differences to calculate an aggregated effect size across studies. Based on CST theory, we expect there to be an increased parasympathetic response.

## Review

Methods

Search Strategy

A meta-analysis of the literature was conducted according to Preferred Reporting Items for Systematic Reviews and Meta-Analyses (PRISMA) guidelines to determine the effect of CST on the ANS as measured by HRV data. Google Scholar, PubMed, and Scientific Direct databases were searched. Articles published in 2007 and beyond were used in the analysis. The research protocol was published on March 13, 2024 [10].

Eligibility Criteria

Articles that reported HF and LF mean and standard deviation data immediately before and after any type of CST were included. Articles that reported HRV data in any follow-up period other than immediately after treatment were excluded. Additionally, any articles published in languages besides English or that did not report pre- vs. post-CST HRV data were not included.

Study Selection

Articles were screened using the aforementioned databases. The following search terms were used, “Craniosacral treatment heart rate variability” and “Neurophysiologic effect of craniosacral treatment”. All entries populated using these search terms were assessed for study qualification. If the article's title contained the relevant topic, the abstract was read for further eligibility. Two independent reviewers selected studies in this research according to the following inclusion and exclusion criteria. Final decisions were made by the principal investigator.

Data Extraction

The data extracted from these articles were HF and LF power pooled means and standard deviations. Data were inputted into Review Manager version 5.4 for analysis. The Review Manager settings included continuous data type, inverse variance statistical method, random effects, and standardized mean difference effect measured with a 95% confidence interval. Standardized mean differences were calculated from these collected data to determine if CST affected HF and LF parameters.

Risk of Bias Assessment

The risk of bias was assessed with a National Institute of Health quality assessment of the case-control study tool (Table 1). Two independent reviewers assessed the studies for bias risk. If the study met all the inclusion, exclusion, and bias risk criteria, the data was extracted for use in this study. These data were inputted into Review Manager version 5.4 to generate a standardized mean difference for each study, summarized into a pooled effect size, and visually displayed on a forest plot. I2 was used to assess heterogeneity. No financial support was obtained for this research. The authors declare no conflicts of interest.

Results

**Figure 1:**
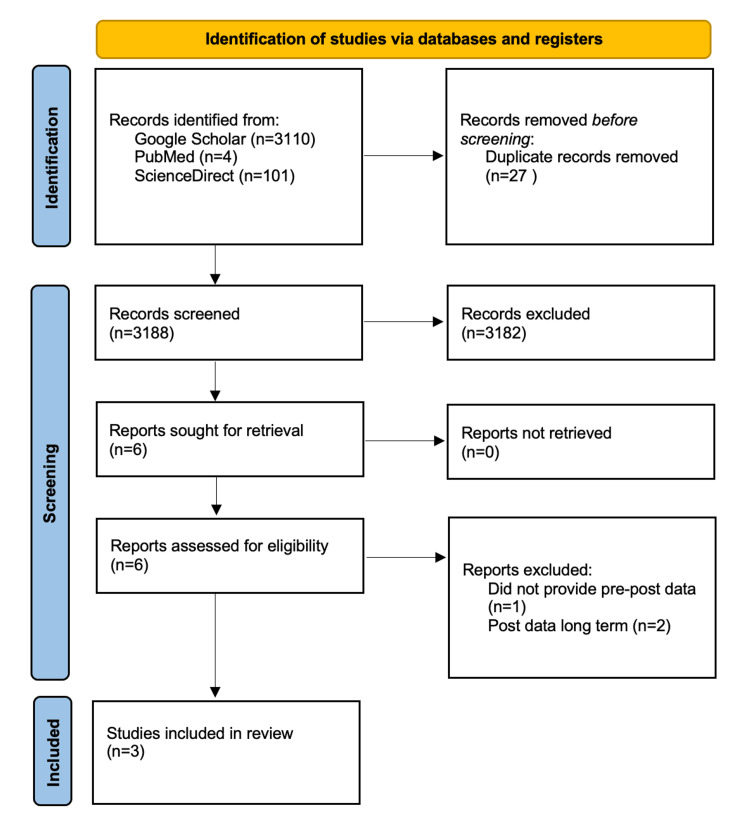
PRISMA 2020 Flow Diagram PRISMA: preferred reporting items for systematic reviews and meta-analysis

**Figure 2:**
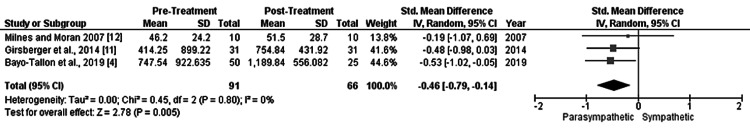
The Effect of CST on HF Power Forest Plot CST: craniosacral therapy; HF: high-frequency

**Figure 3:**
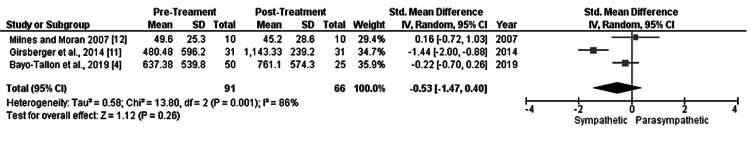
The Effect of CST on LF Power Forest Plot CST: craniosacral therapy; LF: low-frequency

Three articles were included in the meta-analysis of HF and LF power data collected in patients pre-CST and immediately post-CST [4,11-12] (Figure 1). The years of studies ranged from 2007-2019. For both the HF and LF power analyses, a total of 91 patients were analyzed in the pre-treatment group while 66 patients were analyzed in the post-treatment group. The HF power analysis showed a statistically significant negative effect size of pre- vs. post-CST favoring parasympathetic activity; Standardized Mean Difference = -0.46; 95% CI (-0.79,-0.14) (Figure 2). The LF power analysis showed no statistically significant difference in effect size of pre- vs. post-CST; Standardized Mean Difference = -0.53; 95% CI (-1.47, 0.40) (Figure 3). The HF analysis exhibited no heterogeneity with an I2 of 0%. The LF analysis exhibited considerable heterogeneity with an I2 of 86%. For all three studies, the main limitation in the risk of bias analysis was confounding variables were not statistically adjusted for (Table 1).

**Table 1:** NIH Quality Assessment of Case-Control for Risk of Bias Assessment NIH: National Institute of Health

Questions	Milnes and Moran 2007 [12]	Girsberger et al., 2014 [11]	Bayo-Tallón et al., 2019 [4]
Was the research question or objective in this paper clearly stated and appropriate?	Yes	Yes	Yes
Was the study population clearly specified and defined?	Yes	Yes	Yes
Did the authors include a sample size justification?	Yes	Yes	Yes
Were controls selected or recruited from the same or similar population that gave rise to the cases (including the same timeframe)?	Yes	Yes	Yes
Were the definitions, inclusion and exclusion criteria, algorithms, or processes used to identify or select cases and controls valid, reliable, and implemented consistently across all study participants?	Yes	Yes	Yes
Were the cases clearly defined and differentiated from controls?	Yes	Yes	Yes
If less than 100% of eligible cases and/or controls were selected for the study, were the cases and/or controls randomly selected from those eligible?	Yes	Yes	Yes
Was there use of concurrent controls?	No	Yes	Yes
Were the investigators able to confirm that the exposure/risk occurred prior to the development of the condition or event that defined a participant as a case?	Yes	Yes	Yes
Were the measures of exposure/risk clearly defined, valid, reliable, and implemented consistently (including the same time period) across all study participants?	Yes	Yes	Yes
Were the assessors of exposures/risk blinded to the case or control status of participants?	No	Yes	Yes
Were key potential cofounding variables measured and adjusted statistically in the analysis? If matching was used, did the investigators account for matching during study analysis?	No	No	No

Discussion

The association of manual facilitation of the CRI with changes in autonomic function was first hypothesized by Upledger et al. in 1983 [1]. Restriction within the craniosacral system can affect its components: the brain, spinal cord, and protective membranes [13]. Since the initial theory proposal, studies have investigated the validity and effectiveness of CST [7,8]. The theory behind CST postulates that gentle pressure manipulations can stimulate parasympathetic tone and promote ANS balance [3].

To investigate the effect of CST on ANS function, we conducted a meta-analysis of three studies that examined the impact of CST on HRV. The HF power analysis, which corresponds to the changes in parasympathetic activity, revealed a statistically significant negative effect size and thus indicates that CST may lead to an increased parasympathetic response. This aligns with the theory and goals of CST and warrants further investigation. However, it is noteworthy that we found no significant change in the LF power analysis, which reflects both parasympathetic and sympathetic activity.

Some studies were excluded from our meta-analysis due to a lack of consistent methods or reported data values. However, the findings are important to report in the context of our results. Zullow and Reisman found that CV4 and sacral hold/iliac hold treatment led to an increase in parasympathetic activity [14]. Another group of researchers found that both CST and control touch for 5 weeks led to significant changes in heart rate and LF, indicating no difference between the two techniques [15]. Castro- Sánchez et al. found no significant change in HRV values in a study of CST on patients with fibromyalgia [16]. Additionally, a case report of a 39-year-old male patient with post-viral postural orthostatic tachycardia syndrome (POTS) was successfully treated with CST [17]. The authors suggested that POTS induced a hyper-sympathetic state which was reduced with CV4 treatment.

Some limitations must be addressed in future studies to increase validity. One such limitation is highlighted by Bayo-Tallón et al. which centers around the possibility that changes in HRV may derive from the placebo effect of receiving a treatment rather than the facilitation of the CRI itself [4]. Further, the small number of studies included in our meta-analysis and the variation in treatment protocols and patient populations may limit the generalizability of our findings. For example, Milnes and Moran used only the CV4 technique while the other two studies used a combination of cranial techniques including the CV4 technique [4,11-12]. Additionally, we only assessed the short-term effects of CST on HRV, medium and long-term effects will require further studies. Overall, our meta-analysis provides preliminary evidence supporting the potential of CST to modulate parasympathetic activity as evidenced by the HF analysis. Clinically, this suggests there may be an acute benefit of CST in patients with an unbalanced overactive sympathetic state. However, further research utilizing standardized protocols, larger sample sizes, and long-term follow-up is warranted to illustrate its clinical implications for various health conditions.

## Conclusions

The CRI is based on the concept that CSF pulsates indefinitely with a subtle inherent rhythm. CST, a method originating from osteopathy, utilizes this rhythm by manipulation of overlying structures. Anatomical association with the ANS cultivates the theory that such treatment can elucidate autonomic change. The results of this study suggest a statistically significant correlation between osteopathic manipulation of the CRI through CST inducing short-term parasympathetic activity. The aforementioned meta-analysis serves to narrow the gap in supportive evidence of the treatment’s neurophysiological impact. This research supports the recommendation that CST can be used to balance patients’ overactive sympathetic states. Further research is needed to confirm and expand upon these findings, explicate the mechanisms of action, and develop specific protocols for CST in clinical practice.

## References

[1] Upledger JE, Vredevoogd JD (1983). Subtle Energies.

[2] Whedon JM, Glassey D (2009). Cerebrospinal fluid stasis and its clinical significance. Altern Ther Health Med.

[3] Haller H, Lauche R, Sundberg T, Dobos G, Cramer H (2019). Craniosacral therapy for chronic pain: A systematic review and meta-analysis of randomized controlled trials. BMC Musculoskelet Disord.

[4] Bayo-Tallón V, Esquirol-Caussa J, Pàmias-Massana M, Planells-Keller K, Palao-Vidal DJ (2019). Effects of manual cranial therapy on heart rate variability in children without associated disorders: Translation to clinical practice. Complement Ther Clin Pract.

[5] Singh N, Moneghetti KJ, Christle JW, Hadley D, Plews D, Froelicher V (2018). Heart rate variability: An old metric with new meaning in the era of using health technologies for health and exercise training guidance. Part one: Physiology and methods. Arrhythm Electrophysiol Rev.

[6] Hadaya J, Ardell JL (2020). Autonomic modulation for cardiovascular disease. Front Physiol.

[7] Edzard E (2012). Craniosacral therapy: A systematic review of the clinical evidence. Focus Altern Complement Ther.

[8] Green C, Martin CW, Bassett K, Kazanjian A (1999). A systematic review and critical appraisal of the scientific evidence on craniosacral therapy. https://www.ncbi.nlm.nih.gov/books/NBK67710/.

[9] Malik M, Camm AJ (1993). Components of heart rate variability—what they really mean and what we really measure. Am J Cardiol.

[10] Cook AC, Egli A, Cohen NE (2024). Protocol: Neurophysiological effects of craniosacral treatment on heart rate variability. Protocols.io.

[11] Girsberger W, Bänziger U, Lingg G, Lothaller H, Endler PC (2014). Heart rate variability and the influence of craniosacral therapy on autonomous nervous system regulation in persons with subjective discomforts: A pilot study. J Integr Med.

[12] Milnes K, Moran RW (2007). Physiological effects of a CV4 cranial osteopathic technique on autonomic nervous system function: A preliminary investigation. Int J Osteopath Med.

[13] Castro-Sánchez AM, Lara-Palomo IC, Matarán-Peñarrocha GA, Saavedra-Hernández M, Pérez-Mármol JM, Aguilar-Ferrándiz ME (2016). Benefits of craniosacral therapy in patients with chronic low back pain: A randomized controlled trial. J Altern Complement Med.

[14] Zullow M, Reisman S (1997). Measurement of autonomic function during craniosacral manipulation using heart rate variability. Proc IEEE Northeast Bioeng Conf.

[15] Wójcik M, Siatkowski I (2023). The effect of cranial techniques on the heart rate variability response to psychological stress test in firefighter cadets. Sci Rep.

[16] Castro-Sánchez AM, Matarán-Peñarrocha GA, Sánchez-Labraca N, Quesada-Rubio JM, Granero-Molina J, Moreno-Lorenzo C (2011). A randomized controlled trial investigating the effects of craniosacral therapy on pain and heart rate variability in fibromyalgia patients. Clin Rehabil.

[17] Tafler L, Chaudry A, Cho H, Garcia A (2023). Management of post-viral postural orthostatic tachycardia syndrome with craniosacral therapy. Cureus.

