# Correction: The Neurophysiological Effects of Craniosacral Treatment on Heart Rate Variability: A Systematic Review of Literature and Meta-Analysis

**DOI:** 10.7759/cureus.c356

**Published:** 2025-10-13

**Authors:** Andrew C Cook, Anna E Egli, Nathan E Cohen, Kyrie Bernardi, Min Y Chae, Brandon A Kapalko, Sunni A Coyne, Randy Scott

**Affiliations:** 1 College of Osteopathic Medicine, Lake Erie College of Osteopathic Medicine - Bradenton, Jacksonville, USA; 2 College of Osteopathic Medicine, Lake Erie College of Osteopathic Medicine - Bradenton, St. Augustine, USA; 3 College of Osteopathic Medicine, Lake Erie College of Osteopathic Medicine - Bradenton, Daytona Beach, USA; 4 Regional Dean, Lake Erie College of Osteopathic Medicine - Bradenton, Jacksonville, USA

This article has been corrected to fix the following typo in the PRISMA diagram: 'Records excluded' corrected from 0 to 3,182

